# Integrating remote CIED monitoring into heart failure care: evidence, challenges, and opportunities

**DOI:** 10.3389/fcvm.2026.1734567

**Published:** 2026-05-29

**Authors:** Raimondo Calvanese, Carmen D'Amore, Claudio Capobianco, Francesco Di Fraia, Michelangelo Canciello, Bernardino Tuccillo

**Affiliations:** 1 Department of Cardiology, Ospedale del Mare, ASL Napoli 1 Centro, Napoli, Italy; 2 Department of Cardiology, Azienda Ospedaliero Universitaria San Giovanni di Dio Ruggi d'Aragona, Salerno, Italy

**Keywords:** cardiac implantable electronic devices, heart failure, HeartInsight, HeartLogic, remote monitoring, telemedicine, TriageHF

## Abstract

Remote monitoring (RM) of cardiac implantable electronic devices (CIEDs) has evolved from simple device integrity checks to a cornerstone of personalized heart failure (HF) management. By enabling early detection of subclinical deterioration, CIED-based RM supports proactive clinical interventions, potentially reducing hospitalizations and improving outcomes. Multiparametric algorithms such as HeartLogic, TriageHF, and HeartInsight integrate hemodynamic and arrhythmic parameters to predict HF events with good sensitivity. However, despite increasing evidence of clinical and economic benefits, RM implementation remains inconsistent due to heterogeneous protocols, data latency, and inadequate reimbursement structures. This review summarizes current evidence, operational challenges, and future opportunities for integrating remote CIED monitoring into comprehensive HF care pathways, highlighting the role of artificial intelligence and the need for standardized workflows and reimbursement models.

## Introduction

Heart failure (HF) is a clinical syndrome associated with substantial morbidity and mortality and remains one of the leading causes of hospitalization worldwide. Rates of rehospitalization are high: approximately 25%–30% of patients are readmitted within one year after discharge, and up to 50% within three years (1–3). According to data from clinical registries and recent trials, an estimated 10%–15% of patients with severe or advanced HF in the United States and Europe carry a cardiac implantable electronic device (CIED); in the most recent trials in HF with reduced ejection fraction (HFrEF), this prevalence reaches ∼30% (4, 5). In recent years, multiparametric monitoring of patients with CIEDs has enabled both verification of device functional status and early detection of subclinical pathophysiological changes that precede HF worsening.

### Purpose of the review

The objective of this narrative review is to evaluate the current state of the art in remote monitoring for heart failure via Cardiac Implantable Electronic Devices (CIEDs), highlighting the primary distinctions among commercially available algorithms regarding their underlying technology and clinical evidence. Furthermore, this work provides an in-depth analysis of the organizational frameworks and the prevailing barriers to their implementation within the Italian healthcare system. We conducted a research of the Medline database via PubMed, and of the Coachrane Library for studies published between 2010 and 2025, focusing on remote monitoring of heart failure through implantable devices. The search strategy employed a combination of keywords and MeSH terms, including ‘remote monitoring’, ‘heart failure’, and ‘implantable devices’. In addition, a search of the ClinicalTrials.gov registry was performed to identify primary ongoing trials on the subject.

## Management of alerts in patients with CIEDs

Remote monitoring (RM) programs for patients with CIEDs require predefined alert hierarchies and response times. Most alerts involve findings that do not require immediate action (e.g., atrial fibrillation detection), whereas alerts related to device integrity or clinically relevant arrhythmias (e.g., ventricular arrhythmias) necessitate prompt intervention (6). Arrhythmic alerts—including delivered shocks or anti-tachycardia pacing (ATP)—not only indicate increased risk of further arrhythmias but may also signal lead integrity problems.

Each RM center should, based on its local resources, establish and disseminate clear protocols that define: (i) time allocation for daily/weekly alert review, (ii) criteria for patient contact and escalation, and (iii) division of responsibilities within the multidisciplinary team.

**Figure 1:**
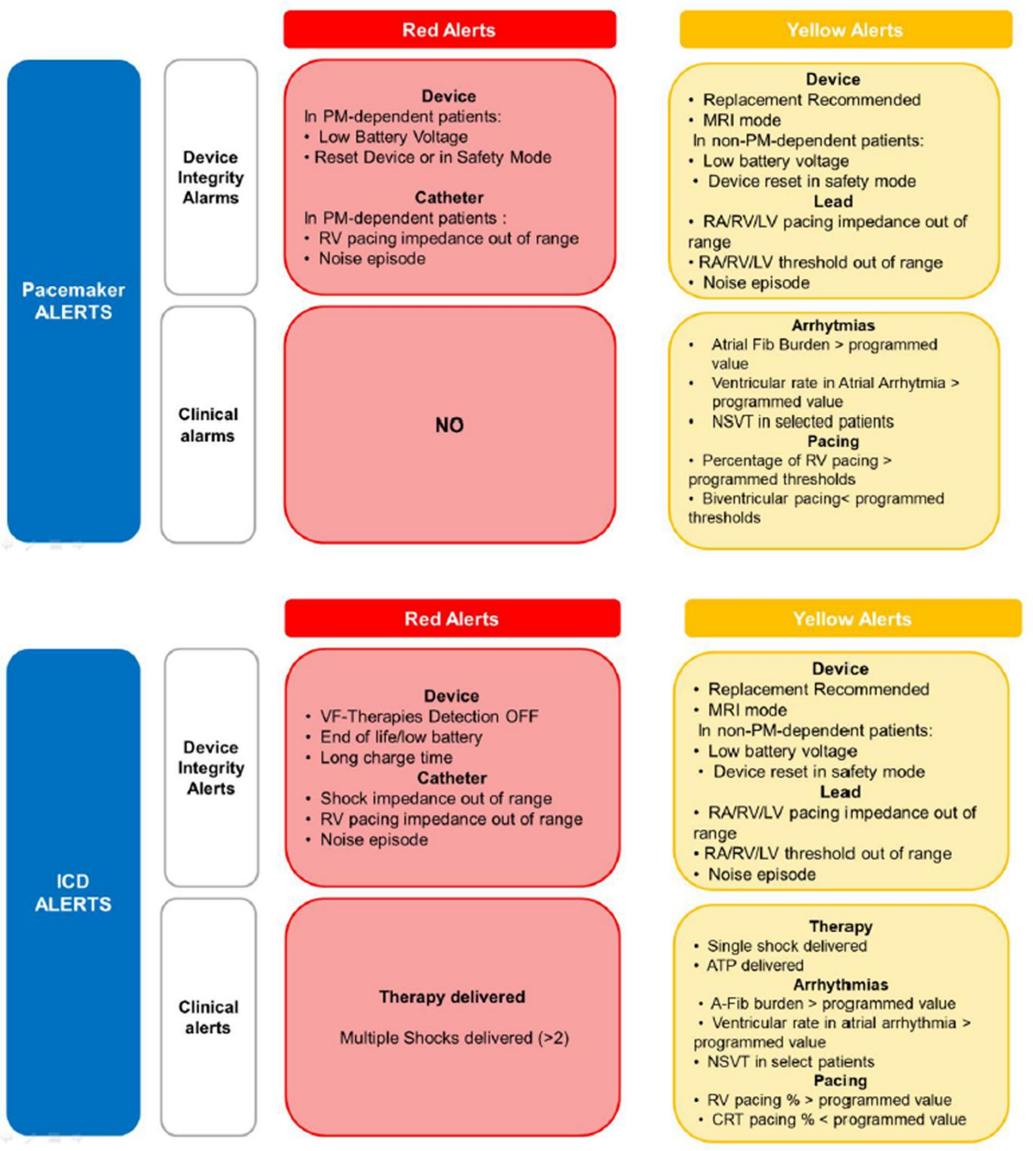
Red (high-priority) and yellow alerts related to clinical events and device integrity in patients with pacemakers and defibrillators. AF, atrial fibrillation; ATP, anti-tachycardia pacing; LV/RV/RA, left/right ventricle/right atrium; NSVT, non-sustained ventricular tachycardia; PM, pacemaker; VF, ventricular fibrillation.

Alerts are commonly categorized into two priority levels (Figure 1): (i) red alerts, generated by warnings concerning device function (e.g., low battery, abrupt impedance changes, shocks), which require rapid intervention; and (ii) yellow alerts, indicating conditions that warrant attention (e.g., gradual increase in capture threshold) and typically allow deferred intervention (Table 1).

**Table 1:** Examples of red and yellow alerts and suggested actions for patients with pacemakers and defibrillators. (See source file for full operational details.).

**PACEMAKER**	
**Red Alerts**	**Actions to take**
Low battery Voltage	Intensify remote controls Schedule PM replacement
PM in Safety Mode (Reset)	In-person check for exit from safety mode
Pacing Impedance out of range	in-person control
Signs of catheter breakage (Noise)	In -person Control Check for EMI (Electromagnetic Interference) EC Breakage: consider implanting a new catheter

**Yellow Alerts**	**Actions to take**
Battery replacement	Schedule replacement in adequate time
MRI Mode	Check exit from MRI mode
Non-PM dependent patients Low voltage battery Reset mode	Intensify checks/schedule Substitution In-person checks
Pacing impedance RA/RV/LV out of range	Check the impedance trend/Possible EC replacement
RA/RV/LV Pacing thresholds out of range	Check pacing threshold trends/Change polarity pacing
Noise episode	Check Impedances/Correlations with EMI/Break/Change polarity pacing
AF burden greater of threshold value	OAT evaluation/ Antiarrhythmic therapy evaluation/ Cardioversion
NON-sustained Ventricular Tachycardia	Clinical evaluation
RV pacing percentage greater than the threshold value	Clinical evaluation A-V delay modification Upgrading to CRT
CRT pacing less than programmed value	Clinical Evaluation Atrial Fibrillation: AV Node Ablation AV Delay Modification High PVC Burden: Antiarrhythmic Therapy/Ablation

DEFIBRILLATORS	
**Red Alerts**	**Recommended Actions**
VT-FV detection OFF	In-person visit in a short time
End of Life/Low Energy	Schedule replacement quickly
Long charging times	Intensify checks/Plan replacement
Shock impedance out of range	Check trends/Intensify checks/Visit in person
RV lead impedance out of range	In-person control:
Noise Episode	In person visit: Check for EMI (Electromagnetic Interference) lead fracture

**Yellow Alerts**	**Recommended Actions**
Replacement recommended	Plan replacement
MRI mode	Check
RA/RV/LV Pacing thresholds out of range	Check trends/Possible EC replacement
Pacing thresholds out of range	Check trends/Programming changes/EC replacement
Single shock delivered	Contact patient/Intensify checks
ATP delivered	Contact patient/Intensify checks
AF burden greater than programmed value	Check OAC indication
Ventricular rate in Atrial Arrhythmia greater than programmed value	Contact patient/Check adherence to therapy/Clinical evaluation
NSVT in selected patients	Intensify checks in relation to the patient
Percentage of RV pacing greater than programmed value	Clinical evaluation (upgrading to CRT/programming changes)
Percentage of biventricular pacing less than programmed value	Clinical evaluation Atrial fibrillation: Antiarrhythmic therapy/PV ablation/AV node ablation PVC: Antiarrhythmic therapy/ablation

Typical responses to device alerts include:

- Calling the patient for a rapid in-clinic evaluation (e.g., repeated shocks, battery depletion, suspected lead fracture);
- Advancing a scheduled outpatient visit according to alert urgency;
- Intensifying remote checks when parameter changes do not require immediate action;
- Tailoring alert programming to the individual patient to avoid unnecessary workload (e.g., disabling atrial fibrillation alerts in patients with permanent AF).

For pacemakers, routine remote transmissions are recommended every 3–12 months; for implantable cardioverter–defibrillators (ICDs), every 3–6 months. As the device approaches elective replacement, transmission frequency should increase to every 1–3 months (6). While RM systems share common principles, they differ substantially in philosophy and practical implementation, including the type and number of programmable alerts.

## Rationale for remote monitoring in heart failure

CIED-based RM in HF supports early detection of clinical and subclinical deterioration, enables proactive and individualized care, may reduce healthcare utilization and costs, and enhances patient engagement. HF is characterized by progressive worsening, increasing hospitalizations, and declining quality of life (7, 8). Each acute decompensation worsens prognosis and is associated with markedly increased short-term mortality and the risk of rehospitalization (9, 10). Given the high rehospitalization burden and the substantial proportion of patients with HFrEF who carry CIEDs (4, 5), a structured CIED-based RM program is an attractive strategy to mitigate risk.

Parameters derived from CIEDs—such as surrogates of fluid status and arrhythmia burden—help clinicians identify trajectories suggestive of impending decompensation, as well as conditions that could precipitate inappropriate shocks or compromise therapies that modify prognosis (e.g., suboptimal biventricular pacing). By detecting changes early, healthcare teams can adjust therapy promptly, potentially preventing hospitalization and improving outcomes.

RM also facilitates more precise, up-to-date assessments of day-to-day status than periodic in-person visits, allowing clinicians to optimize medications, advise on lifestyle, and consider additional interventions based on objective data trends. From an economic perspective, the growing HF burden is straining health systems: in the United States, total HF-related medical costs were projected to rise from US$20.9 billion in 2012 to US$53.1 billion by 2030 (11). In Italy, the average cost per HF admission is ∼€3,200, with an annual burden of ∼€550 million for acute HF admissions (12). By enabling timely interventions and reducing avoidable hospital encounters, CIED-based RM can contribute to cost containment (13).

## Algorithms for remote monitoring of heart failure

Early experiences with CIED-based remote monitoring for HF were largely based on single-parameter strategies, most notably intrathoracic impedance monitoring. The OPTILINK HF trial (14) was one of t the first studies that investigated whether impedance-guided remote monitoring could improve outcomes in patients with chronic HF implanted with ICD or CRT-D devices. In this multicenter, randomized trial, automatic alerts were generated when predefined impedance thresholds were crossed, prompting clinical review and potential therapeutic intervention. However the study didn't show a significant reduction in the primary composite endpoint of all-cause mortality or cardiovascular hospitalization compared with standard care. These neutral results were likely influenced by the limited specificity of impedance as a standalone marker of congestion, variability in alert-driven clinical responses across centers, and the multifactorial pathophysiology of HF decompensation, which cannot be adequately captured by a single physiological signal. Importantly, the OPTILINK experience highlighted the limitations of single-sensor monitoring strategies and provided the basis for the development of contemporary multiparametric algorithms, by integrating congestion surrogates with autonomic, arrhythmic, and activity-related parameters. These new systems allow to improve predictive accuracy, reduce false-positive alerts, and enhance clinical interpretability, thereby enabling more timely and targeted interventions.

**Figure 2:**
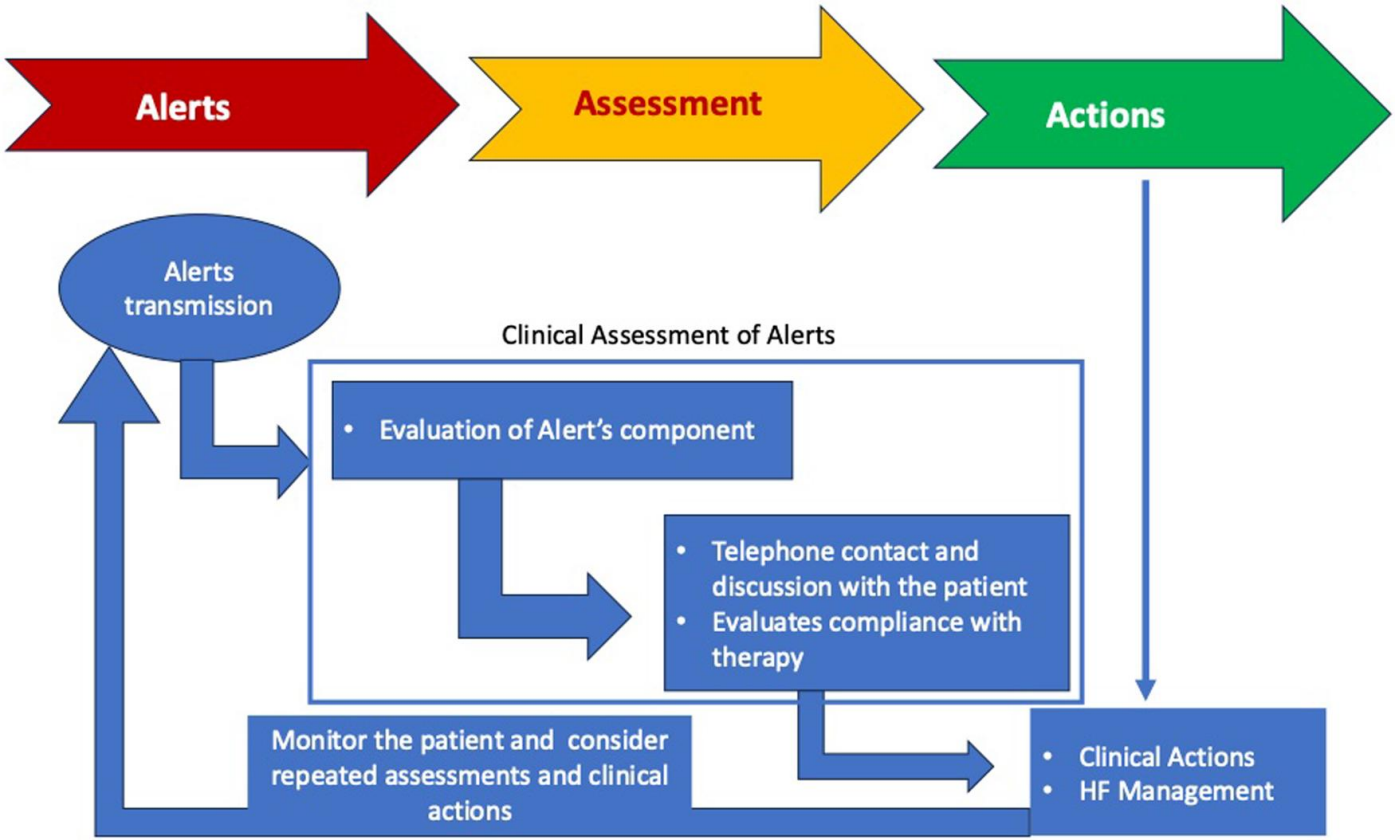
Suggested algorithm for the management of an HF alert generated by CIED-based remote monitoring.

Three commonly used multiparametric algorithms support early detection of HF events: HeartLogic (15, 16), TriageHF (17, 18), and HeartInsight (derived from SELENE-HF) (19–21). These tools integrate multiple device-derived signals into a composite index that correlates with short-term HF risk, enabling proactive outreach (Figure 2). Patients in an alert state have higher risk of HF events, atrial fibrillation events (19), and mortality (20).

Typical sensing inputs include first and third heart sounds (S1/S3), respiration rate and rapid shallow breathing index, thoracic impedance, day- and night-time heart rate, patient activity, ventricular ectopy, and atrial tachyarrhythmia burden. These sensors can capture HF pathophysiology before patients perceive symptoms.

Operationally, when a composite index exceeds a programmed threshold, an alert is sent to the RM team (e.g., via email/SMS). Clinicians review the dashboard to identify the dominant contributors and, after clinical triage, contact the patient to assess symptoms, adherence, and potential triggers, and to adjust therapy or behaviors as appropriate.

Subsequent remote checks allow rapid assessment of the response to the intervention without an excessive workload increase.

Physiological and device-derived inputs include first and third heart sound (S1/S3), respiratory rate, day and night heart rate trends, patient activity, atrial arrhythmia burden, and thoracic impedance. These sensors can capture HF pathophysiology before patient perceive symptoms.

Depending on the system, they may generate a clinical alert, when the composite index exceeds a programmed threshold, or classify patients into predefined risk stratification to indicate potential clinical worsening.

- HeartLogic (Boston Scientific): combines S3/S1 heart sounds, thoracic impedance, activity level, night heart rate, and respiratory rate into a single index. A nominal threshold of 16 is commonly used to balance sensitivity and specificity; crossing the threshold indicates elevated risk of an HF event within ∼34 days, with ∼70% sensitivity (16, 19). When the heartLogic index crosses the threshold, an alert was notify to the center. Alert review provides transparency on sensor contributions to guide targeted actions (e.g., optimizing decongestive therapy when thoracic impedance trends suggest fluid accumulation).
- HeartInsight (Biotronik; implementation following SELENE-HF): the score integrates baseline clinical parameters with temporal trends in seven monitored variables (24-hr and nocturnal mean heart rate, atrial fibrillation burden, daily activity, heart rate variability, ventricular extrasystoles, and thoracic impedance). An automatic alert is generated when three consecutive transmissions yield a score ≥ the nominal threshold (NT = 45). The alert resets when the score falls below a recovery threshold (NT−10). An NT of 45 is recommended as the default (21).

Triage-HF (Medtronic): combines several physiological and device-derived signals, including nocturnal heart rate, patient physical activity, burden of atrial tachyarrhythmias, heart rate trends, and thoracic impedance to generate a stratified risk score (low, medium, or high) to identify early signs of clinical worsening

## Management of the HF alert: are we aligned?

Despite the promise of RM, real-world implementation remains heterogeneous, and evidence-based, scalable follow-up protocols are limited. Programs differ in timing and modality of patient contact, criteria for remote therapy adjustment, use of natriuretic peptide testing, and scheduling of in-person assessments. While BNP/NT-proBNP have established roles in HF prognostic stratification and dyspnea evaluation, their role in longitudinal monitoring is debated due to conflicting data: in HFrEF, rapid BNP increases may not predict exacerbations as reliably as in HF with preserved EF (HFpEF). Nevertheless, changes in natriuretic peptides can complement CIED alerts and enhance positive predictive value (16, 22, 23). When feasible, obtaining NT-proBNP in association with specific HF alerts and comparing to baseline may increase protocol sensitivity, recognizing that device-based indices can change well before symptoms.

Importantly, recent evidence suggests that the clinical benefit of RM depends less on the monitoring technology itself and more on the presence of timely, structured clinical reactions to alerts. As showed in a *post hoc* analysis of OPTILINK study (24), remote monitoring was associated with improved outcomes only when alerts triggered appropriate medical action, including therapy optimization and clinical reassessment. In that study, patients who were appropriately contacted following an alert experienced a significant reduction in the primary endpoint of cardiovascular death or first hospitalization [hazard ratio, 0.61 (95% CI, 0.39–0.95); *P* = 0.027] compared with those receiving usual care o not appropriate responce. Thus, passive monitoring without organized reaction pathways appears to provide limited benefit. In this context, two studies have specifically explored alert-triggered therapy adjustments.

These studies have explored remote, alert-triggered therapy adjustments. In the MANAGE-HF study (25), 200 patients with hFrEF and cardiac resynchronization therapy or/and implantable cardioverter implants were followed up using the HeartLogic algorithm (Boston Scientific). Early intervention, triggered by alerts, led to a rapid reduction in the HeartLogic index and a decrease in NTproBNP values (1316 pg/mL baseline vs. 1316 pg/mL 12-month visit, *p* < 0.001). Although the early intervention consisted of a medication augmentation (primarily increased diuretic doses) in 74% of cases, the study highlighted the high heterogeneity of intervention protocols, which varied by center and patient. This procative intervention was associated with a 67% reduction in hHF compared to prestudy rates (Hernandez AF, Albert NM, Allen LA et al. Multiple cardiac sensors for management of heart failure (MANAGE-HF) Phase I results. Abstract presented/published at: ESC-HF 2021. June 29-July 1, 2021. Virtual.).

In INTERVENE-HF (18), TriageHF-guided outreach prompted brief diuretic uptitrations with frequent normalization of thoracic impedance. Across studies, diuretic optimization is the most common immediate intervention. *Although the search for robust scientific evidence remains ongoing, it is essential to acknowledge that the significant pathophysiological heterogeneity underlying acute heart failure—coupled with the diverse clinical profiles of patients with varying cardiac etiologies—makes it challenging to standardize a universal therapeutic protocol.*

*The widespread adoption of remote monitoring for heart failure via CIEDs faces significant challenges that, at present, remain unresolved. The implementation of organizational algorithms, their reimbursement, and subsequent clinical application are contingent upon health policies rooted in robust scientific evidence. Currently, such evidence remains elusive, particularly regarding ‘hard' endpoints—such as mortality reduction—which are difficult to demonstrate due to the extensive heterogeneity of both available technological tools and the structural organization of individual healthcare facilities.* Organizational gaps further limit impact. Community-based care integration is often lacking, despite its potential to improve adherence and relieve central RM teams.

Variability across regions and countries complicates standardization and, critically, reimbursement policies remain inconsistent and often inadequate, impeding comprehensive RM adoption.

## Obstacles to the diffusion of RM in Italy

RM is considered standard of care for CIED follow-up in many contemporary recommendations (26). However, rapid growth in RM enrollment—accelerated during the COVID-19 pandemic—has strained staffing and workflows without commensurate reimbursement in many European jurisdictions and Italian regions (27–29). Even where expert consensus provides pragmatic recommendations (6), gaps persist in enrollment coordination, maintenance of connectivity, and device programming for optimal RM (30). Alert interpretation models—particularly for HF alerts—are not standardized, and inconsistent response strategies have yielded conflicting outcomes (31, 32). Data latency introduces further bias: availability at the monitoring center may lag device detection due to connectivity issues or delays in review and escalation (33). Late RM activation and suboptimal connectivity exacerbate the problem and perpetuate reliance on in-person visits (29, 34). A structured workflow—preferably a centralized monitoring unit with trained staff operating a hub-and-spoke model—can improve outcomes (33, 35). Despite heterogeneous use (more frequent in ICD/CRT recipients) and uneven reimbursement (e.g., Germany), RM is generally cost-saving compared with exclusive in-clinic follow-up (36). In Europe, lack of adequate reimbursement remains the principal barrier—reported by up to 40% of centers post-COVID-19 and by up to 73% in Italy—while only 10 of 20 Italian regions currently provide tariffs for remote CIED management [(26, 34); Table 2].

**Table 2:** Overview of reimbursement for RM across European countries, including tariffs for in-clinic checks, remote CIED management, dedicated hardware/services, and HF disease management.

Country	Reimbursement tariff for in-clinic device check	Reimbursement tariff for remote CIED management	Reimbursement specific for hardware and services for remote monitoring	Reimbursement tariff for HF disease management
Austria	Y	N	N	Y
Belgium	Y	N	N	N
Bulgaria	N	N	N	N
Czech Republic	Y	Y	Y	N
Denmark	Y	Y	N	N
Finland	Y	Y	Y	N
France	Y	Y	Y	N
Germany	Y	Y (only ICDs and CRTs)	Y (for some health ensurances)	N
Hungary	Y	Y	N	N
Italy	Y	Y (in 10 out of 20 regions)	N	N
Norway	Y	Y	N	N
Poland	N	N	N	N
Portugal	Y	Y	N	Y
Russia	N	N	N	N
Slovakia	N	N	N	N
Spain	Funded, no tariff	Funded, no tariff	N/A	N
Sweden	Y	Y	N	N
Switzerland	Y	Y	Y	Y
The Netherlands	Y	Y	N	Y
UK	Y	Depending on Clinical Commissioning Groups and NHS Trusts	Ordered by NHS Trusts	N

## Toward a new era in the management of HF

Over the past two decades, RM of CIEDs has evolved from intermittent device integrity checks to continuous, AI-enabled analysis of arrhythmic and hemodynamic parameters. Multiparametric scores predict short-term risk of HF events with reasonable sensitivity (e.g., ∼70%), but unexplained alert rates remain non-trivial (15). Augmenting baseline risk stratification (e.g., Seattle HF Model) and applying AI-based post-processing may reduce false alerts (19). At the same time, optimal clinical response strategies are still being defined. In MANAGE-HF (25), clinicians assessed triggers within three days and escalated therapy as needed—most commonly diuretic intensification, but also guideline-directed medical therapy (GDMT) optimization (e.g., ARNI, ACEi/ARB, MRA, SGLT2i) when appropriate (37). Early decongestive escalation shortened alert duration, whereas systematic GDMT uptitration was less consistently implemented.

Given the robust benefits of disease-modifying agents—often evident within 10–20 days (38, 39)—clinicians should consider prioritizing GDMT optimization when alerts occur in the absence of overt congestion. A recent meta-analysis reported that device-guided, congestion-based management reduced the composite of all-cause mortality and HF hospitalization vs. standard care (40).

Importantly, RM represents an opportunity in elderly patients management (41). In fact RM is associated with high adherence rates, reduced need for in-person visit and allows an early detection on actionable events. However, specific challenges emerged including cognitive impairment, caregiver dependence e higher comorbidity burden, factors that may influence alert transmission and interpretation and therapeutic responsiveness. Thus, it is crucial to develop strategies to enhance the use of RM in this vulnerable group, implementing these approaches in individuals with neurocognitive disorders and visual or hearing impairments continues to present significant challenges. All these challenges could potentially be easly addressed with the support of artificial intelligence.

Economic evaluations, including the randomized EVOLVO trial (42) and subsequent studies (43, 44), showed that RM reduces urgent visits and overall in-hospital encounters, with shorter decision delays after device-detected events—likely contributing to fewer visits and lower costs. *According to a recent systematic review of clinical, cost-effectiveness quality of life and cost outcomes, an advantage in terms of cost-effectiveness has been proved for HeartLogic and TriageHF, compared to the respective cardiac implantable electronic device without these algorithms, though available evidence was often of low quality (45).*

Future research should focus on pragmatic trials that test standardized, alert-driven care pathways; quantify the incremental value of AI vs. clinician-led triage; and rigorously evaluate cost-effectiveness and reimbursement models, particularly in the rapid growing very elderly HF population.

## References

[1] Abdin A Anker SD Butler J Coats AJS Kindermann I Lainscak M. “Time is prognosis” in heart failure: time-to-treatment initiation as a modifiable risk factor. ESC Heart Fail. (2021) 8(6):4444–53. 10.1002/ehf2.13646 34655282 PMC8712849

[2] Metra M Teerlink J. Heart failure. Lancet. (2017) 390:1981–95. 10.1016/S0140-6736(17)31071-1 28460827

[3] Arrigo M Jessup M Mullens W Reza N Shah AM Sliwa K. Acute heart failure. Nat Rev Dis Primers. (2020) 6:16. 10.1038/s41572-020-0151-7 32139695 PMC7714436

[4] Packer M Anker SD Butler J Filippatos G Pocock S Carson P. Cardiovascular and renal outcomes with empagliflozin in heart failure. N Engl J Med. (2020) 383:1413–24. 10.1056/NEJMoa2022190 32865377

[5] McMurray JJ Solomon SD Inzucchi SE Køber L Kosiborod MN Martínez FA. Dapagliflozin in patients with heart failure and reduced ejection fraction. N Engl J Med. (2019) 381:1995–2008. 10.1056/NEJMoa1911303 31535829

[6] Ferrick AM Raj SR Deneke T Kojodjojo P Lopez-Cabanillas N Abe H. 2023 HRS/EHRA/APHRS/LAHRS expert consensus statement on practical management of the remote device clinic. Europace. (2023) 25(5):euad123. 10.1093/europace/euad123 37208301 PMC10199172

[7] Chaudhry SP Stewart GC. Advanced heart failure: prevalence, natural history, and prognosis. Heart Fail Clin. (2016) 12(3):323–33. 10.1016/j.hfc.2016.03.001 27371510

[8] Murphy SP Ibrahim NE Januzzi JL Jr. Heart failure with reduced ejection fraction: a review. JAMA. (2020) 324(5):488–504. 10.1001/jama.2020.10262 32749493

[9] Osenenko KM Kuti E Deighton AM Pimple P Szabo SM. Burden of hospitalization for heart failure in the United States: a systematic literature review. J Manag Care Spec Pharm. (2022) 28(2):157–67. 10.18553/jmcp.2022.28.2.157 35098748 PMC10373049

[10] Blumer V Mentz RJ Sun JL Butler J Metra M Voors AA. Prognostic role of prior HF hospitalization among patients hospitalized for worsening chronic HF. Circ Heart Fail. (2021) 14(4):e007871. 10.1161/CIRCHEARTFAILURE.120.007871 33775110 PMC9990499

[11] Ziaeian B Fonarow GC. Epidemiology and aetiology of heart failure. Nat Rev Cardiol. (2016) 13(6):368–78. 10.1038/nrcardio.2016.25 26935038 PMC4868779

[12] Maggioni AP Spandonaro F. Lo scompenso cardiaco acuto in italia. G Ital Cardiol. (2014) 15(2 Suppl 2):3S–4. 10.1714/1465.16179 24770482

[13] Chen J Wilkoff BL Choucair W Cohen TJ Crossley GH Johnson WB. Design of the pacemaker remote follow-up evaluation and review (PREFER) trial. Trials. (2008) 9:18. 10.1186/1745-6215-9-18 18387185 PMC2311273

[14] Bohm M Drexler H Oswald H Rybak K Bosch R Butter C. Fluid status telemedicine alerts for heart failure: a randomized controlled trial. Eur Heart J. (2016) 37(41):3154–63. 10.1093/eurheartj/ehw099 26984864

[15] Boehmer JP Hariharan R Devecchi FG Smith AL Molon G Capucci A. A multisensor algorithm predicts HF events in patients with implanted devices: multiSENSE. JACC Heart Fail. (2017) 5(3):216–25. 10.1016/j.jchf.2016.12.011 28254128

[16] Gardner RS Singh JP Stancak B Nair DG Cao M Schulze C. Heartlogic identifies periods of increased risk of HF events: multiSENSE. Circ Heart Fail. (2018) 11(7):e004669. 10.1161/CIRCHEARTFAILURE.117.004669 30002113

[17] Virani SA Sharma V McCann M Koehler J Tsang B Zieroth S. Prospective evaluation of integrated device diagnostics for HF management: TRIAGE-HF. ESC Heart Fail. (2018) 5(5):809–17. 10.1002/ehf2.12309 29934976 PMC6165932

[18] Zile MR Costanzo MR Ippolito EM Zhang Y Stapleton R Sadhu A. Intervene-HF: feasibility of risk-stratified intervention in HFrEF. ESC Heart Fail. (2021) 8(2):849–60. 10.1002/ehf2.13231 33527654 PMC8006696

[19] D’Onofrio A Solimene F Calò L Calvi V Viscusi M Melissano D. SELENE-HF: combining home monitoring trends and baseline risk to predict HF hospitalization. Europace. (2022) 24(2):234–44. 10.1093/europace/euab170 34392336 PMC8824514

[20] Bertini M Vitali F Santini L Tavoletta V Giano A Savarese G. ICD-Detected HF status predicts AF occurrence. Heart Rhythm. (2022) 19(5):790–7. 10.1016/j.hrthm.2022.01.020 35066184

[21] Zanotto G Capucci A. Heartinsight from SELENE-HF to clinical practice. Eur Heart J Suppl. (2023) 25(Suppl C):C337–43. 10.1093/eurheartjsupp/suad030 37125280 PMC10132563

[22] Maisel A Barnard D Jaski B Frivold G Marais J Azer M. Primary results of the HABIT trial. J Am Coll Cardiol. (2013) 61(16):1726–35. 10.1016/j.jacc.2013.01.052 23500322

[23] Lewin J Ledwidge M O’Loughlin C McNally C McDonald K. Clinical deterioration in established HF: value of BNP and weight gain. Eur J Heart Fail. (2005) 7(6):953–7. 10.1016/j.ejheart.2005.06.003 16227134

[24] Wintrich J Pavlicek V Brachmann J Bosch R Butter C Oswald H. Remote monitoring with appropriate reaction to alerts was associated with improved outcames in chronic heart failure: results from the OpriLink HF study. Circ Arrhythm Electrophysiol. (2021) 14(1):e008693. 10.1161/CIRCEP.120.008693 33301362

[25] Hernandez AF Albert NM Allen LA Ahmed R Averina V Boehmer JP. Phase I evaluation of HeartLogic in HF. J Card Fail. (2022) 28(8):1245–54. 10.1016/j.cardfail.2022.03.349 35460884

[26] Simovic S Providencia R Barra S Kircanski B Guerra JM Conte G. RM During COVID-19: EHRA physician survey. Europace. (2022) 24(3):473–80. 10.1093/europace/euab215 34410364 PMC8499732

[27] Fulchand S. COVID-19 and cardiovascular disease. Br Med J. (2020) 369:m1997. 10.1136/bmj.m1997 32434891

[28] Magnocavallo M Bernardini A Mariani MV Piro A Marini M Nicosia A. Home delivery of communicators for RM during lockdown. Pacing Clin Electrophysiol. (2021) 44(6):995–1003. 10.1111/pace.14251 33908052 PMC8207054

[29] Boriani G Burri H Svennberg E Imberti JF Merino JL Leclercq C. Reimbursement practices for RM across Europe. Europace. (2022) 24(12):1875–80. 10.1093/europace/euac118 35904006 PMC9384581

[30] Bertini M D’Onofrio A Piacenti M Lavalle C La Greca C Amellone C. Real-world practice versus RM recommendations for CIEDs. Heart Rhythm O2. (2024) 6(3):246–52. 10.1016/j.hroo.2024.11.024 40201678 PMC11973669

[31] Kolk MZH Narayan SM Clopton P Wilde AAM Knops RE Tjong FVY. Long-term mortality reduction with RM in ICD patients. Europace. (2023) 25:969–77. 10.1093/europace/euac280 36636951 PMC10062290

[32] Calò L Bianchi V Ferraioli D Santini L Dello Russo A Carriere C. Multiparametric ICD algorithm for HF risk in practice. Circ Heart Fail. (2021) 14(10):e008134. 10.1161/CIRCHEARTFAILURE.120.008134 34190592 PMC8522625

[33] Husser D Christoph Geller J Taborsky M Schomburg R Bode F Nielsen JC. Information flow and workflow in IN-TIME. Eur Heart J Qual Care Clin Outcomes. (2019) 5(2):136–44. 10.1093/ehjqcco/qcy031 30016396 PMC6440440

[34] Maines M Palmisano P Del Greco M Melissano D De Bonis S Baccillieri S. Impact of COVID-19 on RM in Italy: AIAC survey. J Clin Med. (2021) 10(18):4086. 10.3390/jcm10184086 34575197 PMC8469719

[35] Zanotto G Melissano D Baccillieri S Campana A Caravati F Maines M. Intrahospital model for RM data sharing: AIAC document. J Cardiovasc Med (Hagerstown). (2020) 21(3):171–81. 10.2459/jcm.0000000000000912 32004241

[36] Ricci RP Vicentini A D’Onofrio A Sagone A Rovaris G Padeletti L. Economic analysis of RM: TARIFF study. Heart Rhythm. (2017) 14(1):50–7. 10.1016/j.hrthm.2016.09.008 27614025

[37] Vaduganathan M Claggett BL Jhund PS Cunningham JW Ferreira JP Zannad F. Lifetime benefits of comprehensive therapies in HFrEF. Lancet. (2020) 396(10244):121–8. 10.1016/S0140-6736(20)30748-0 32446323

[38] Sinagra G Pagura L Stolfo D Fabris E Savarese G Rapezzi G. Combining new drug classes for HFrEF. Eur J Intern Med. (2021) 90:10–5. 10.1016/j.ejim.2021.05.017 34090751

[39] Rosano GMC Vitale C Adamo M Metra M. Roadmap for management during the vulnerable phase post-HF hospitalization. J Cardiovasc Med (Hagerstown). (2022) 23(3):149–56. 10.2459/JCM.0000000000001221 34937849 PMC10484190

[40] Zito A Princi G Romiti GF Galli M Basili S Liuzzo G. Device-based RM strategies for congestion-guided HF care: systematic review and meta-analysis. Eur J Heart Fail. (2022) 24(12):2333–41. 10.1002/ejhf.2655 36054801 PMC10086988

[41] Scacciavillani R Koliastasis L Doundoulakis I Chiotis S Kordalis A Narducci ML. Remote monitoring of cardiac implantable electronic devices in very elderly patients: advantages and specific problems. J Cardiovasc Dev Dis. (2024) 11(7):209. 10.3390/jcdd1170209 39057629 PMC11277150

[42] Landolina M Perego GB Lunati M Curnis A Guenzati G Vicentini A. EVOLVO: RM reduces healthcare use in HF with ICDs. Circulation. (2012) 125(24):2985–92. 10.1161/CIRCULATIONAHA.111.088971 22626743

[43] Boriani G Da Costa A Ricci RP Quesada A Favale S Iacopino S. MORE-CARE phase 1: dynamics of early intervention with RM. J Med Internet Res. (2013) 15(8):e167. 10.2196/jmir.2608 23965236 PMC3758044

[44] Klersy C Boriani G De Silvestri A Mairesse GH Braunshweig F Scotti V. Effect of telemonitoring of CIEDs on healthcare utilization: meta-analysis. Eur J Heart Fail. (2016) 18(2):195–204. 10.1002/ejhf.470 26817628

[45] Kenny R Bhattarai N O'Connor N Gonzalez-Moral SG O'Keefe H Hosseini-Jebeli S. Algorithm-based remote monitoring of heart failure risk data in people with cardiac implantable electronic devices: a systematic review and cost-effectiveness analysis. Health Technol Assess. (2025) 29(50):1–160. 10.3310/PPOH2916 PMC12666606 41108091

